# Genetically Encoded Whole Cell Biosensor for Drug Discovery of HIF-1 Interaction Inhibitors

**DOI:** 10.1021/acssynbio.2c00274

**Published:** 2022-10-12

**Authors:** Louis H. Scott, Mark J. Wigglesworth, Verena Siewers, Andrew M. Davis, Florian David

**Affiliations:** † Discovery Sciences, Biopharmaceuticals R&D, AstraZeneca, SE-41320 Gothenburg, Sweden; ‡ Discovery Sciences, Biopharmaceuticals R&D, AstraZeneca, Alderley Park SK10 2NA, U.K.; § Department of Biology and Biological Engineering, Division of Systems and Synthetic Biology, Chalmers University of Technology, SE-41296 Gothenburg, Sweden; ∥ Discovery Sciences, Biopharmaceutical R&D, AstraZeneca, Cambridge, CB2 0AA, U.K.

**Keywords:** drug discovery, biosensor, new modality, genetic circuit, directed evolution, high-throughput screen

## Abstract

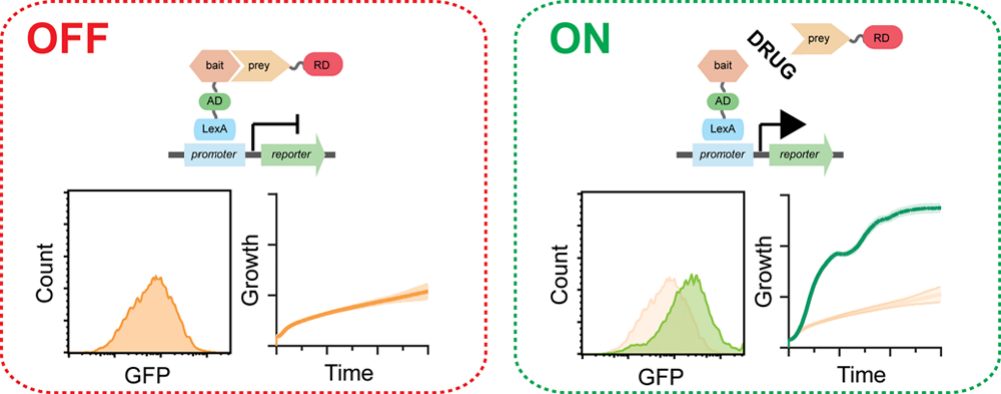

The heterodimeric transcription factor, hypoxia inducible factor-1 (HIF-1), is an important anticancer target as it supports the adaptation and response of tumors to hypoxia. Here, we optimized the repressed transactivator yeast two-hybrid system to further develop it as part of a versatile yeast-based drug discovery platform and validated it using HIF-1. We demonstrate both fluorescence-based and auxotrophy-based selections that could detect HIF-1α/HIF-1β dimerization inhibition. The engineered genetic selection is tunable and able to differentiate between strong and weak interactions, shows a large dynamic range, and is stable over different growth phases. Furthermore, we engineered mechanisms to control for cellular activity and off-target drug effects. We thoroughly characterized all parts of the biosensor system and argue this tool will be generally applicable to a wide array of protein–protein interaction targets. We anticipate this biosensor will be useful as part of a drug discovery platform, particularly when screening DNA-encoded new modality drugs.

## Introduction

HIF-1 is a heterodimeric transcription factor composed of HIF-1α and HIF-1β subunits that activates expression of genes for angiogenesis, glucose metabolism, cell proliferation, and metastasis in response to hypoxia.^1^ HIF-1 is overexpressed in many cancers, leading to intratumoral adaptation to hypoxia and resistance to cancer treatments.^1^ Manipulation of HIF-1 expression has shown beneficial effects on tumor growth, making it a good target to drug in combination therapies.^2^ Nevertheless, few traditional small molecule drug discovery efforts have provided leads against HIF-1 dimerization,^3^ let alone generated clinically available drugs.^4^ The importance of HIF-1 in cancers, compounded with difficulties in finding specific inhibitors, provides a strong impetus to explore alternative drug discovery approaches to use against HIF-1.

New modality therapeutics, such as peptides, RNA therapeutics, protein degraders and antibody conjugates offer an innovative solution against hard to hit targets, such as protein–protein interactions (PPIs) like HIF-1.^5^ Discovery of these biology-based therapeutics can be supported by synthetic biology approaches, where living systems are engineered to not only produce new chemical diversity but also to discern functionality using genetic screens.^6^ While assays for drug discovery against HIF-1 have been engineered in different mammalian cell lines,^7,8^ their use is limited when compared to implementations in simpler organisms such as bacteria or yeasts that can also be efficiently engineered to display tens of millions of new modality therapeutic variants. For example, engineered bacteria were used to screen libraries of genetically encoded cyclic peptides against a variety of PPI targets, including HIF-1.^9−11^

The yeast *Saccharomyces cerevisiae* has advantages over bacteria as a drug discovery tool. It is evolutionarily closer to humans, meaning any finding should translate better into the clinic;^12^ yeast can glycosylate proteins, which enables it to mimic human targets with better fidelity;^13^ and efforts are underway to humanize yeast.^14^ Yeast can also produce a wide array of compounds, and many characterized genetic parts can be combined to screen a variety of molecules for drug leads.^6^

Testament to the power of yeast, it is the archetypical system for studying PPIs using the yeast two-hybrid system (YTH).^15^ Here, if two proteins interact, they induce the expression of a reporter gene by creating an artificial transcription factor. Similarly, the HIF-1 transcription factor has been expressed in yeast and shown to activate a reporter gene downstream of its cognate binding motif.^16^ While this assay was used to identify HIF-1 effectors, it is neither versatile nor robust. It cannot be adapted to investigate other PPIs as it requires cognate HIF-1 transcriptional activation and DNA binding. More fundamentally, the YTH system is not suited to discover PPI inhibitors as it lacks a positive selection, as desired compounds do not cause reporter gene expression.

A clever variation of the yeast two-hybrid system, the repressed transactivator (RTA) yeast two-hybrid biosensor system, was engineered as a drug discovery screen to identify small molecule protein interaction inhibitors.^17,18^ Here, when two proteins interact, they repress the expression of a reporter gene. The reporter gene is only then expressed if a compound prevents the PPI, thus providing a positive selection for PPI inhibition and making the selection for potential false positives less likely. The RTA system however relies on growth as readout, which is difficult to precisely quantify, thus making ranking of relative drug potency inaccurate. Furthermore, no mechanisms to control for drug off-target effects are in place, leading to potential false-positives that could quickly overgrow a culture if growth is used as selection. Addressing these issues should provide a more robust assay, thus reducing potential drug candidate attrition.

We wanted to expand the RTA system as a versatile yeast-based drug discovery tool and then validate it using HIF-1 as the target. To do this, we explored the use of fluorescence as reporter output to enable fluorescent activated cell sorting (FACS) to be used in conjunction with growth selection, which is more suitable as a prescreening step. Additionally, we thoroughly characterized all individual parts in the sensor, and where possible, we used orthogonal systems to those endogenous to yeast. Furthermore, we engineered an internal control for biosensor cell health and an additional control strain to detect false positives. Using our optimized RTA system, we show fluorescence output and growth selection for the biosensor strain when HIF-1α/HIF-1β dimerization is inhibited, establishing this new tool as an important element in future drug discovery platforms.

## Results

### Design and Construction of a Protein–Protein Interaction Inhibitor Biosensor with Fluorescence Output

**Figure 1:**
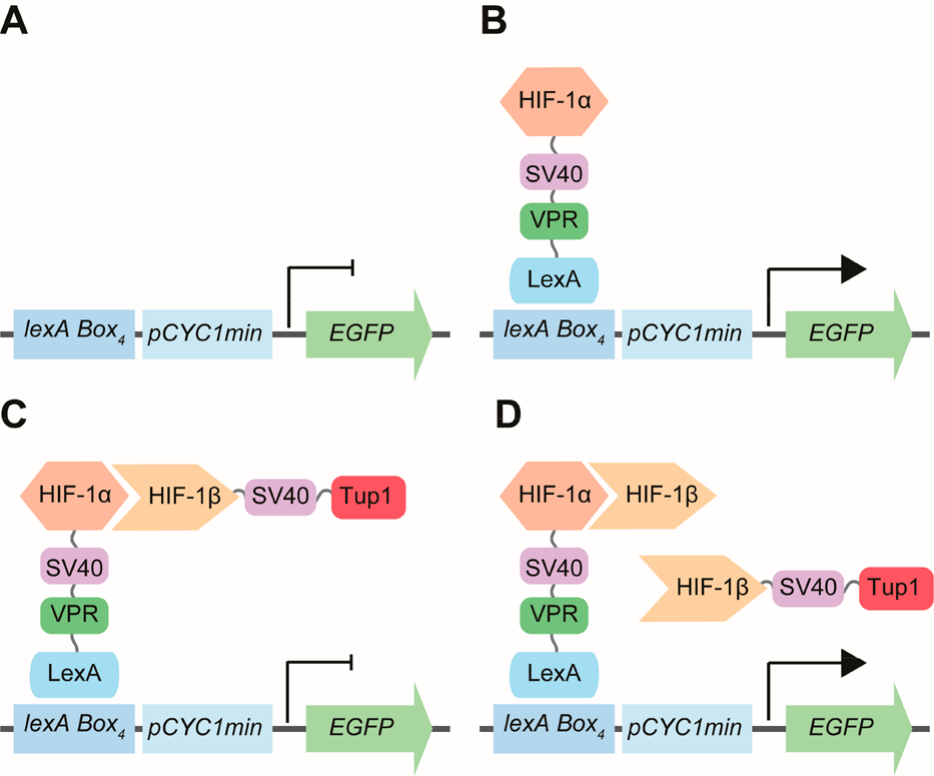
Representation of the repressed transactivator two-hybrid system adapted to detect inhibitors of HIF-1α/HIF-1β dimerization by fluorescence. (A) In the absence of the transactivator, the reporter gene (*EGFP*) is not transcribed. (B) Fusion of bait protein (HIF-1α) with the transactivator (LexA-VPR-SV40) causes expression of EGFP via interaction with *lexA* operators (*lexA Box*) upstream of the minimal *CYC1* promoter. (C) A fusion of the transcriptional repressor (Tup1-SV40) and prey protein (HIF-1β) will interact with the bait (HIF-1α) and cause repression of EGFP expression. (D) Inhibition of HIF-1α/HIF-1β dimerization (e.g., via an additional HIF-1β subunit) causes reporter derepression and results in expression of EGFP. SV40 = nuclear localization sequence (NLS).

A schematic describing the expanded RTA system designed in this study specifically to detect HIF-1α/HIF-1β dimerization inhibitors is found in Figure 1. Briefly, a transcriptional activator fused to a bait protein (HIF-1α) will induce expression of a reporter gene when targeted to it via DNA binding domains. When a prey protein (HIF-1β) is coexpressed as a fusion to a transcriptional repressor, it will turn off the reporter gene through bait–prey interaction. The reporter gene is only then turned back on when a compound (e.g., small molecule) inhibits the bait–prey PPI. Details describing specific parts redesigned in this study are outlined in the following sections.

**Figure 2:**
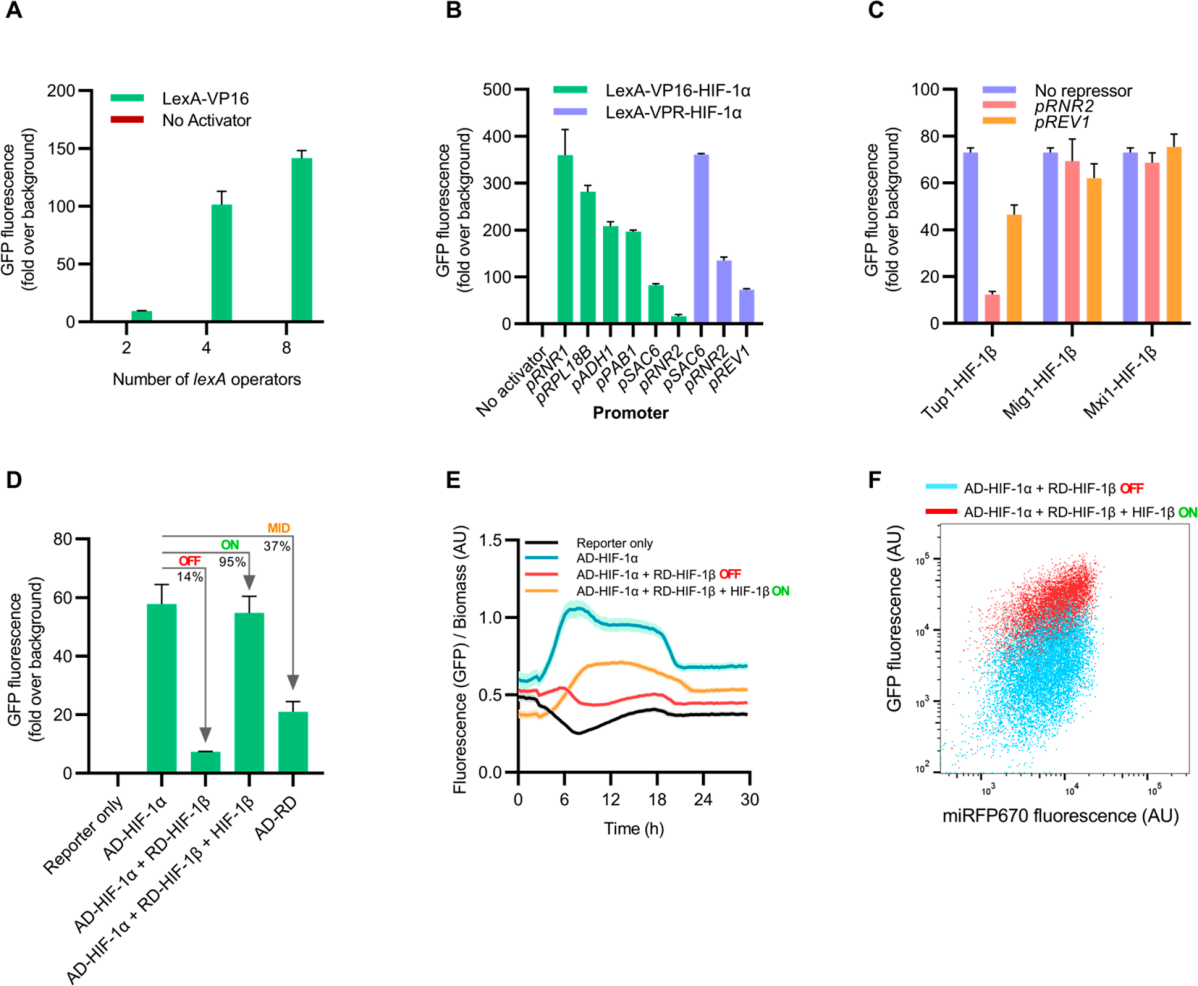
Design and construction of a protein–protein interaction biosensor with fluorescence output. (A) Fluorescence (GFP) output from reporter genes (with either 2, 4, or 8 *lexA* operators) with or without coexpression of the LexA-VP16 transactivator from the *ADH1* promoter. (B) Fluorescence (GFP) output from the reporter gene *lexAx4-pCYC1min-EGFP* in cells coexpressing transcriptional activators (either LexA-VP16 or LexA-VPR) fused to the bait protein HIF-1α. Transcriptional activator–bait fusions were expressed from different promoters as denoted on the *x*-axis. (C) Transcriptional repression of the reporter gene *lexAx4-pCYC1min-EGFP* in cells coexpressing the transactivator–bait (LexA-VPR-HIF-1α) from the *REV1* promoter, along with various repressor–prey proteins (Tup1-HIF-1β, Mig1-HIF-1β, or Mxi1-HIF-1β) as denoted on the *x*-axis. Repressor–prey proteins were expressed from either the *RNR2* or *REV1* promoter. (D) Dynamic range of the biosensor. Fluorescence (GFP) output is shown as maximal reporter gene (*lexAx4-pCYC1min-EGFP*) output (AD-HIF-1α), repressed “OFF” state output (AD-HIF-1α + RD-HIF-1β), and derepressed “ON” state output (AD-HIF-1α + RD-HIF-1β + HIF-1β). Biosensor derepression is achieved when an additional HIF-1β subunit is coexpressed. Additionally, fluorescence output from the reporter gene *lexAx4-pCYC1min-EGFP* in cells coexpressing the transactivator-repressor fusion (AD-RD) from the *REV1* promoter is shown as a midway “MID” state. The output of the sensor in either “OFF”, “ON”, or “MID” states is shown as a percentage of the original biosensor fluorescence. (E) Time course study of the dynamic range of the biosensor. Fluorescence output relative to biomass for strains described in panel D was measured over time. Bars represent mean values, and error bars represent the standard deviation. (F) Fluorescence (GFP) output of the biosensor reporter gene (*lexAx4-pCYC1min-EGFP*) relative to constitutive miRFP670 expression. GFP fluorescence of the biosensor strain in the “OFF” state (AD-HIF-1α + RD-HIF-1β) increases relative to miRFP670 fluorescence in the “ON” state when coexpressing an additional HIF-1β subunit (AD-HIF-1α + RD-HIF-1β + HIF-1β). For panels A-D, fluorescence was measured by flow cytometry after 16 h of culture; bars represent mean values, and error bars represent the range. For panels D–F, AD = LexA-VPR, RD = Tup1, and AD-RD = LexA-VPR-Tup1.

We chose the enhanced green fluorescent protein (EGFP)^19^ as a FACS-compatible reporter gene. Additionally, we chose the bacterial DNA-binding protein LexA^20^ to interact with the reporter gene promoter as an orthogonal solution to the *GAL* system used in the previous setup.^17^ Using these parts, we explored whether a genetic configuration could lead to high reporter gene expression, yet would also be sensitive to transactivation. We genomically integrated cassettes with either two, four, or eight *lexA* operators^21^ upstream of the minimal *CYC1* promoter (*pCYC1min*) to drive EGFP expression (*lexAx2-pCYC1min-EGFP*, *lexAx4-pCYC1min-EGFP*, or *lexAx8-pCYC1min-EGFP*). In these strains we also coexpressed a LexA-based^22^ transactivator comprised of the full length LexA repressor which binds the *lexA* operators upstream of the reporter gene, the SV40 nuclear localization signal (NLS)^23^ to ensure transport of the fusion into the nucleus, and the strong transcriptional activator VP16.^24^ The transactivator cassette was also genomically integrated and expressed from the constitutive *ADH1* promoter. GFP fluorescence was measured by flow cytometry to evaluate reporter gene activation (Figure 2A). EGFP expression was seen only in cells coexpressing the LexA-NLS-VP16 fusion and increased with number of *lexA* binding boxes. However, the intensity of the fluorescence induction saturated when more than four *lexA* boxes were present. These results show the reporter gene with four operator sequences is the best performer when considering both fluorescence output and responsiveness to the transactivator.

We subsequently investigated if the transactivator could still activate gene expression as a tripartite fusion displaying a bait protein. We also explored reporter gene expression when the transactivator–bait fusion was expressed from weak or strong promoters characterized in the modular cloning (MoClo) yeast tool kit (YTK).^25^ Additionally, we tested the use of a different transcriptional activator; while VP16 is a powerful transcriptional activator,^21^ VPR, which is a fusion of three different activation domains (VP64, p65, and Rta38), is considerably stronger.^26^ We integrated cassettes expressing either LexA-NLS-VP16 or LexA-NLS-VPR fused to the bait HIF-1α and measured their ability to activate *EGFP* (*lexAx4-pCYC1min-EGFP*) expression via flow cytometry. We found that despite fusion of the bait protein, transactivators containing either VP16 or VPR provided a strong fluorescence signal (Figure 2B). Furthermore, the VPR activator was strong enough to induce a signal even when expressed from the weakest promoters in the YTK collection (i.e., *pREV1*). As the biosensor should be most responsive to inhibitors when its parts are least abundant,^17^ we continued biosensor testing with the strain expressing the transactivator–bait fusion from the *REV1* promoter.

We next investigated if a PPI could recruit a transcriptional repressor to our reporter gene, thus turning it off. As HIF-1 is a heterodimer of HIF-1α/HIF-1β, we expressed HIF-1β as prey while fused to an NLS and either of the transcriptional repressors: Tup1 (N-terminal repression domain),^18,27^ Mig1,^28^ or Mxi1 (N-terminal repression domain).^29,30^ We expressed these repressor fusions from either the *RNR2* or *REV1* promoters in the reporter strain (*lexAx4-pCYC1min-EGFP)* coexpressing the transactivator–bait (LexA-VPR-HIF-1α) from the *REV1* promoter. When measuring reporter gene fluorescence by flow cytometry (Figure 2C), we found Tup1 to provide the best transcriptional repression, and that repression increased with repressor–prey expression. These results indicate that repression of reporter gene expression is caused by bait–prey interaction.

### Validation of the Fluorescent RTA Biosensor

From our results characterizing the reporter gene, transactivator–bait, and repressor–prey fusions, we deemed a system with four *lexA* operators (*lexAx4-pCYC1min-EGFP*), expressing the VPR transactivator-HIF-1α fusion from the *REV1* promoter, and the Tup1 repressor-HIF-1β fusion from the *RNR2* promoter would be a suitable biosensor configuration. With this configuration, the biosensor is in a repressed “OFF” state and only outputs 14% of the maximal fluorescence when compared to reporter gene transactivation solely from LexA-VPR-HIF-1α (Figure 2D). We next needed to validate if inhibition of HIF-1α/HIF-1β dimerization could be registered as an increase in fluorescence output from this “OFF” state. We expressed an additional HIF-1β subunit from a centromeric plasmid using the *TEF1* promoter in this biosensor strain. We observed derepression of the reporter gene by flow cytometry, presumably caused by competitive binding of the additional HIF-1β subunit to the transactivator-HIF-1α (Figure 2). The sensor in this “ON” state returned to 95% of the maximal fluorescence output when compared to reporter gene transactivation solely from LexA-VPR-HIF-1α (Figure 2D). As the HIF-1α/HIF-1β Per-Arnt-Sim (PAS) B domain interaction *K*_D_ is 378 nM,^31^ the sensor should therefore be sensitive enough to detect inhibitors close to this potency.

Our previous results imply that the reporter gene is sensitive to the interplay between transactivator–bait and repressor–prey expression. As these parts are driven by endogenous promoters that may up- or down-regulate throughout different growth stages in a batch cultivation, we did a time course study to measure the fluorescence response of the biosensor to an additional HIF-1β subunit. We found the biosensor to be most responsive from 6 to 18 h, during which the largest differences in fluorescence between “ON” and “OFF” states were recorded (Figure 2E). Overexpression of the additional HIF-1β subunit caused a slight growth defect (Figure S3); however, this did not translate into lower relative fluorescence (Figure S4).

It is possible that reporter gene expression in the biosensor is repressed through overexpression of Tup1 and not because of recruitment of the repressor–prey protein. To investigate this, we replaced wild-type HIF-1α with a dimerization weak mutant, HIF-1α_mut_ (Q320E, V336E, and Y340T),^32^ as transactivator–bait fusion in the biosensor strain. This transactivator should only weakly recruit the repressor–prey fusion, resulting in less reporter gene repression. We found the biosensor with mutant HIF-1α displayed significantly less reporter gene repression (39% of the maximal output) when compared to the wild-type transactivator-HIF-1α strain (14% of the maximal output) (Figures S1 and 2D). We next removed the HIF-1α bait from the transactivator fusion in the biosensor strain (while still retaining expression of the repressor-HIF-1β fusion). Although reporter gene expression decreased slightly (only down to 71% of the maximal output) in this variant, this decrease was significantly less than observed when both interacting bait and prey fusions were present (down to 14% of the maximal output) (Figures S2 and 2D). Together, these results indicate reporter gene expression is predominately dependent on PPI and that inhibition of PPI can be selected for using fluorescence.

### Biosensor Controls for Biological and off-Target Effects

The biosensor output could be affected by biological distortions related to cell size and growth, or by off-target and pleiotropic effects from screened drugs. To mitigate this, we integrated a cassette expressing miRFP670^33^ under control of the constitutive *TEF1* promoter to provide a red fluorescence signal to normalize the GFP output of the biosensor strain with. Derepression of this biosensor from “OFF” to “ON” states, achieved by coexpressing an additional HIF-1β subunit, is observed as an increase in GFP expression relative to miRFP670 (Figure 2F).

In parallel, we engineered an additional biosensor strain to counter-screen against false-positive signals that could arise if a drug has off-target effects, for example by binding to the repressor and inactivating it. In this strain, the protein-interacting partners were omitted, leaving a fusion of LexA-VPR-Tup1. When recruited to the *lexA*-controlled reporter gene, the signal strength is midway “MID” between the fully activated and fully repressed biosensor, achieving only 37% of the maximal reporter gene output (Figure 2D). Here, if a drug inactivates the repressor, thus giving a false positive signal in the primary screen, we should be able to discern this as an increase in fluorescence.

### Design and Construction of a Protein–Protein Interaction Inhibitor Biosensor with Auxotrophic Selection

The original RTA sensor system uses auxotrophy as selection, a powerful way to quickly screen large libraries without limitations of FACS throughput. Unfortunately, auxotrophic output is generally binary, where cells grow or not, making the relative assessment of selected inhibitor potency difficult. We reasoned instead that auxotrophic selection would be better suited as a prescreening step used in conjunction with FACS to decrease the number of library members before more detailed investigation of potency via fluorescence.

**Figure 3:**
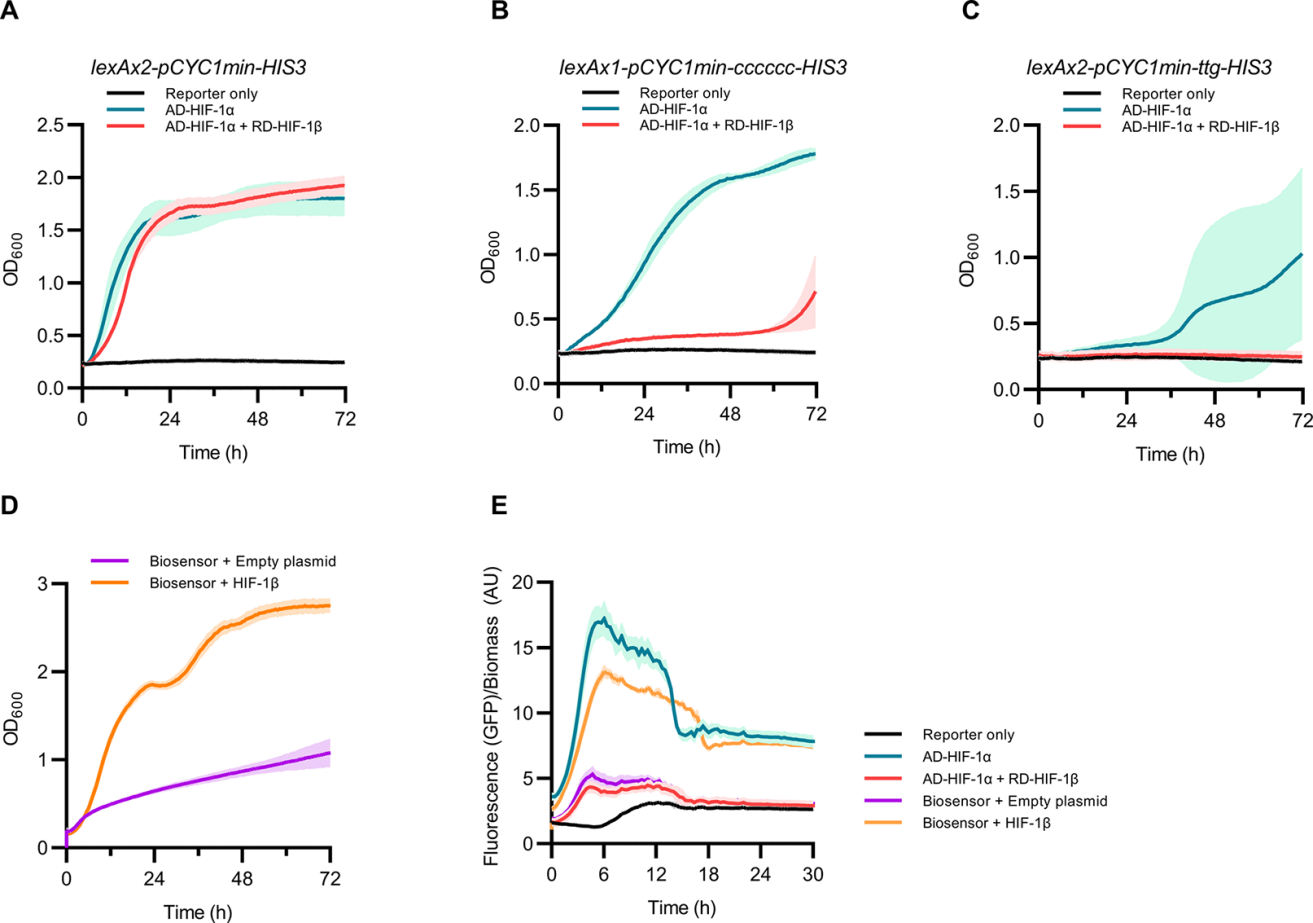
Construction and validation of a protein–protein interaction biosensor with fluorescence output and auxotrophic selection. (A–C) Yeast strains containing either the *lexAx4-pCYC1min-EGFP* reporter (reporter only), or also the *pREV1-LexA-VPR-HIF-1α* transactivator–bait (AD-HIF-1α), or also both transactivator–bait and the *pRNR2-TUP1-HIF-1β* repressor–prey (AD-HIF-1α + RD-HIF-1β) were grown in media lacking histidine (SD-H) when also harboring *HIS3* reporter variants. (D) The biosensor strain also containing the *lexAx1-pCYC1min-cccccc-HIS3* reporter gene was grown in media lacking histidine and uracil (SD-H-U) when harboring an empty plasmid or one expressing an additional HIF-1β subunit. (E) Time course measurement of fluorescence output per biomass for strains harboring the *lexAx1-pCYC1min-cccccc-HIS3* reporter gene and other parts that make up the biosensor. Yeast strains were grown in SD or SD-U media. For panels A–D, yeast strains were grown in 96-well microtiter plates using a growth profiler (EnzyScreen). For panel E, yeast strains were grown in a 48-well FlowerPlate in a BioLector (m2p-laboratories GmbH). Bars represent mean values, and error bars represent the standard deviation.

To take advantage of growth selection as a prescreen step, we integrated a cassette expressing *HIS3* from the minimal *CYC1* promoter controlled by two *lexA* operators (*lexAx2-pCYC1min-HIS3*) into histidine auxotrophic yeast strains also engineered with various parts of the biosensor (combinations of reporter gene, transactivator–bait, and repressor–prey). We found the strain expressing the *HIS3* reporter, additional to the *EGFP* reporter and the transactivator–bait, grew similarly to the strain also coexpressing the repressor–prey, indicating that the *HIS3* reporter gene was not repressed enough to prevent growth in media lacking histidine (Figure 3A).

We made additional variations of the *HIS3* reporter gene in an effort to lessen leaky expression based on strategies to decrease transcription and/or translation.^34^ First, we retained the two *lexA* boxes in the reporter gene promoter region but replaced the native *HIS3* ATG start codon with the rare TTG variant (*lexAx2-pCYC1min-ttg-HIS3*). We also engineered a reporter gene with only a single *lexA* operator and mutated the Kozak consensus region^35^ to hexa-cytosine (*lexAx1-pCYC1min-cccccc-HIS3*). We found the strain with the TTG start codon could not survive in media lacking histidine when only the transactivator–bait was coexpressed, indicating *HIS3* expression was too low in this variant (Figure 3C). We found however that the variant with one *lexA* box and the deoptimized Kozak sequence grew in media lacking histidine when only coexpressing the transactivator–bait yet did not survive when also coexpressing the repressor–prey (Figure 3B). This result suggests that the *HIS3* gene is repressed enough to allow selection for HIF-1α/HIF-1β dimerization inhibitors.

To validate the auxotrophic selection, we expressed an additional HIF-1β subunit from a centromeric plasmid using the *TEF1* promoter in the biosensor strain also harboring the *lexAx1-pCYC1min-cccccc-HIS3* reporter gene. We observed derepression of both fluorescent and auxotrophic reporter genes (Figure 3D,E), indicating both selection mechanisms were functional within the same strain.

## Discussion

As protein-based new modality drugs can be DNA encoded, it stands to reason to use cells as living factories to produce them, enabling the display of tens of millions of drug variants simply by expressing a DNA library. However, this strategy is limited by traditional high-throughput screening protocols that can assay only hundreds of thousands of variants. A solution is to use an internal selection mechanism within every drug-producing cell, enabling the producer cell to also decipher whether it has made a functional drug variant. Such a selection would need to meet throughput demands yet also provide a sensitive readout related to potency. This work presents an addition to the genetic selection toolbox intended to address these issues.

Fundamental to any engineering discipline, the biosensor presented is modular, easy to engineer, allows rapid prototyping, and behaves in a predictable manner. We designed and built the optimized RTA biosensor with a plug-and-play mindset by adopting the yeast modular cloning toolkit assembly method.^25^ Biosensor parts can be easily combined to express many target variants, at differing expression levels, when fused to transcriptional activators and repressors of choice. Furthermore, recent toolkit developments facilitate these constructs to be directly integrated into desired genomic locations.^36^ We also observed a clear relationship between part and function (e.g., transcriptional activator strength and fluorescence signal), giving predictability to the system as well as future-proofing it. Our system should therefore be open to new parts as they become characterized. Future work should explore other transcriptional repressors, possibly by combining repression domains,^37^ as we did not see strong repression using Mxi1 or Mig1 as reported elsewhere.^28,29^ More efficient repression of the system would provide a larger dynamic range. Ultimately, statistical modeling could inform part choice for finely tuned sensors intended for specific applications.^38^

The biosensor provides an output that is stable, tunable, able to be interpreted easily with general laboratory methods, and with high dynamic and operational range. It could discern between strong and weak interacting partners, making the system a useful way to select for relative drug potency. While the biosensor could detect a nM binder (HIF-1β subunit PASB domain), it was not fully activated (albeit close to 100%), suggesting the limit of detection may be lower. Future work should relate binding kinetics with sensor output^39^ to better gauge sensor sensitivity. Additionally, existing cyclic peptide inhibitors with known potency against HIF-1 should be expressed in the sensor strain to ascertain limits of detection^10^ and would be an important first step should this sensor be used to display peptide variants. Ultimately this assay is more intended as a primary screen from which the most potent hits would be channeled into a secondary biophysical assay for further characterization.

The sensor is also stable, likely owing to the genomic integration of all biosensor parts, whereas previous iterations mostly used episomal expression.^17^ Furthermore, it has a large operational window, important as an inhibitor made *in vivo* may need time to accumulate within the cell to a functional concentration. Care should be taken to express any genetically encoded modalities using a promoter similar to, or stronger than, the *TEF1* promoter (used to express the HIF-1β subunit during validation) to ensure accumulation of therapeutics to a functional concentration.

While fluorescence output is a relative way to measure potency, retaining auxotrophic selection was an important design choice. High-efficiency yeast transformation enables the construction of libraries with 10^10^ members,^40^ a number greater than can be practically screened by FACS. Magnetic-activated cell sorting is a solution for this in antibody display projects;^41^ we argue, however, that library diversity can be significantly lowered using a far simpler life-or-death prescreening strategy that does not require additional laborious materials or protocols. By limiting auxotrophic selection to a prescreening step, the potential growth of false-positive or escape mutants that could overgrow the culture of desired drug variants is restricted.

A major advantage our system offers is the ability to normalize the output signal to cellular activity or health. While a drug may have a negative effect on cell health, it may still bind the intended target. In such a scenario, the GFP output may be low, yet this would be considered a positive signal if it was high relative to miRFP670. Such a hit could offer a functional starting point from which medicinal chemistry could optimize specificity.

An obstacle with any screening strategy is overcoming off-target effects that produce a false-positive signal. Particularity for hard to hit targets, where the interface of protein dimers may not contain many druggable pockets, the likelihood of selecting an off-target hit might indeed be more probable. For example, this could be a hit binding the repressor and rendering it nonfunctional, or binding the activator and increasing its activity. To mitigate this risk, the control biosensor strain would act as a counter-screen to use against short-listed hits from the primary screen. Having a counter-screen in a separate strain also controls for biosensor escape mutants. Here, a nonsense mutation in the repressor or a mutation in one of the targets making them weakly interact will be registered as a positive signal in the primary screen, but as a negative signal in the secondary screen. Interestingly, the biosensor with bait and prey interaction showed greater reporter gene repression than the control biosensor. This difference could be explained by steric hindrance where the HIF-1 dimer might shield the activation domain from recruiting coactivators.

In conclusion, we have engineered a variation of the RTA biosensor system to enable both fluorescence and auxotrophic selections. The biosensor is constructed in a modular fashion, meaning it should be applicable to other PPI targets of interest. Additionally, we provide an option to control for off-target effects and the ability to screen for false-positives. We anticipate this biosensor will be useful as part of a drug discovery platform, particularly when screening DNA-encoded new modality drugs.

## Materials and Methods

### Bacterial Strains and Growth

All plasmids were cloned and amplified in competent DH5α *Escherichia coli* cells. *E. coli* was cultured in lysogeny broth (LB) (10 g/L tryptone, 5 g/L yeast extract, 5 g/L NaCl, 5 mL/L 1 M Tris–HCl, and 20 g/L agar for solid media) and supplemented with appropriate antibiotics (ampicillin 100 mg/mL or chloramphenicol 34 mg/mL). *E. coli* was cultured at 37 °C, with shaking at 200 rpm for liquid cultures.

### Yeast Strains and Growth

*Saccharomyces cerevisiae* CEN.PK 102-5B (*MATa ura3-52 his3Δ1 leu2-3/112 MAL2-8*^*c*^*SUC2*)^42^ was used as background for biosensor strain construction. *S. cerevisiae* was routinely cultured on yeast peptone dextrose (YPD) medium (10 g/L yeast extract, 20 g/L peptone from casein, 20 g/L glucose, and 20 g/L agar for solid media) supplemented with appropriate antibiotics (Nourseothricin, 100 mg/L and Geneticin, 200 mg/L). Synthetic complete dextrose (SD) media (6.7 g/L yeast nitrogen base without amino acids, 20 g/L glucose, 0.79 g of complete supplement mixture, and 20 g/L agar for solid media) was used to maintain plasmids in yeast. Depending on selection, uracil or histidine were omitted. Yeast was cultured at 30 °C and with shaking at 220 rpm for liquid cultures. Yeast was routinely transformed by chemical transformation following the lithium acetate protocol.^43^

### Growth Profiler

Time course measurement of yeast growth was monitored using a growth profiler (EnzyScreen). Yeast strains were inoculated from 24 h precultures to an OD_600_ 0.1 in 250 μL of media. Cultures were grown at 30 °C with shaking at 250 rpm in a 96-well microtiter plate. A minimum of three biological replicates were used per measurement. Data analysis and figure creation were done with GraphPad Prism software.

### Plasmid and Strain Construction and Verification

Plasmids were constructed using either Gibson assembly (Gibson Assembly Master Mix, New England Biolabs), Golden Gate assembly, or restriction ligation as per published protocols.^25,44^ Routine DNA sequence verification was done by Eurofins Genomics. Plasmid and gel extraction kits from ThermoFisher Scientific were used to purify DNA for cloning. Polymerase chain reactions (PCR) were performed using Phusion DNA polymerase (ThermoFisher Scientific) for cloning. Yeast codon optimized genes were synthesized at either GenScript, Integrated DNA Technologies, or TWIST bioscience. Details of plasmids constructed in this study can be found in the supplementary sequences and strains file in the Supporting Information.

The EasyClone-MarkerFree strategy was used to genomically integrate expression cassettes into yeast.^44^ Integrative cassettes were either prepared as per the EasyClone-MarkerFree strategy or amplified by PCR from plasmids with oligonucleotides found in Table S1 that provide homology to genomic sites. Genomic DNA was extracted from yeast by boiling colonies in 20 nM NaOH for 5 min, and 0.5 μL was used as a template to confirm genomic integrations by colony PCR as per the EasyClone-MarkerFree strategy,^44^ or as template for plasmid parts. Colony PCR was performed using SapphireAmp as per the manufacturer’s protocol. The genotype of strains constructed in this study can be found in the supplementary sequences and strains file in the Supporting Information.

### Fluorescence Measurement

Real-time fluorescence was measured using a BioLector (m2p-laboratories GmbH, Baesweiler, Germany). Yeast strains were inoculated from 24 h old precultures to a starting OD_600_ of 0.1 in 1 mL of media in 48-well FlowerPlates. GFP expression was measured as a ratio of GFP fluorescence to biomass. A minimum of three biological replicates were used per measurement. Data analysis and figure creation was done with GraphPad Prism software.

Flow cytometry was undertaken using a Guava easyCyte 8HT system (Merck Millipore). Yeast strains were inoculated from 24 h old precultures to a starting OD_600_ of 0.1 in 250 μL of media in a 96-well microtiter plate and grown for 16 h in a growth profiler (EnzyScreen). Yeast cultures were then diluted to an OD_600_ of 0.02 in 200 μL of water and fluorescent proteins excited with a 488 nm laser (for GFP) or 648 nm laser (for miRFP670). 5000 events were recorded per sample. A minimum of three biological replicates were used per measurement. Fold over background fluorescence was calculated as the fold change in median fluorescence compared to the background yeast strain CEN.PK 102-5B. Data analysis and figure creation was done with GraphPad Prism and FlowJo software.

## Supporting Information Available

biosensor output with dimerization weak or absent protein targets (Figures S1 and S2); growth curves for biosensor strains (Figure S3); fluorescence output of biosensor strains (Figure S4); oligonucleotides used in this study (Table S1) (PDF)
Genotype and sequences of strains constructed (XLSX)
