# Supplementary material for: Genetically Encoded Whole Cell Biosensor for Drug Discovery of HIF-1 Interaction Inhibitors
Source: ACS Synth Biol. 2022 Oct 12;11(10):3182–9. doi: 10.1021/acssynbio.2c00274 (PMC9594322; doi:10.1021/acssynbio.2c00274)
Supplement: Supplementary file 1 — sb2c00274_si_001.pdf [file sb2c00274_si_001.pdf]

Supporting information

Genetically encoded whole cell biosensor for drug discovery of HIF-1 interaction inhibitors

Louis H. Scott[1,3], Mark J. Wigglesworth[2], Verena Siewers[3], Andrew M. Davis[4] and Florian David[3]*

[1]Discovery Sciences, Biopharmaceuticals R&D, AstraZeneca, SE-41320 Gothenburg, Sweden
[2]Discovery Sciences, Biopharmaceuticals R&D, AstraZeneca, Alderley Park, SK10 2NA, UK
[3]Department of Biology and Biological Engineering, Division of Systems and Synthetic Biology, Chalmers University of Technology, SE-41296 Gothenburg, Sweden
[4]Discovery Sciences, Biopharmaceutical R&D, AstraZeneca, Cambridge, CB2 0AA, UK
*Corresponding author; Email: davidfl@chalmers.se.

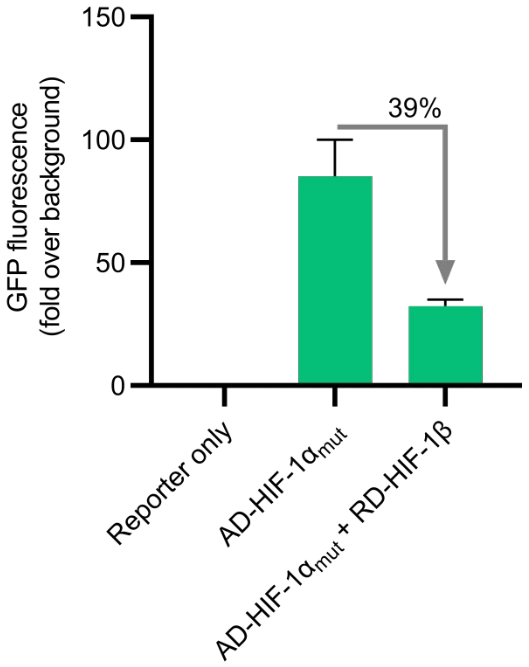

Figure S1. Biosensor reporter gene repression with weakly-interacting HIF-1α protein subunit mutant. Fluorescence (GFP) output is shown as maximal reporter gene (*lexAx4-pCYC1min-EGFP*) output (AD-HIF-1α_{mut}) compared to the repressed state (AD-HIF-1α_{mut} + RD-HIF-1β). Only 39% of the maximal fluorescence is achieved in the repressed state. Fluorescence was measured by flow cytometry after 16 h of culture, and bars represent mean values and error bars the range. AD = LexA-VPR and RD = Tup1.

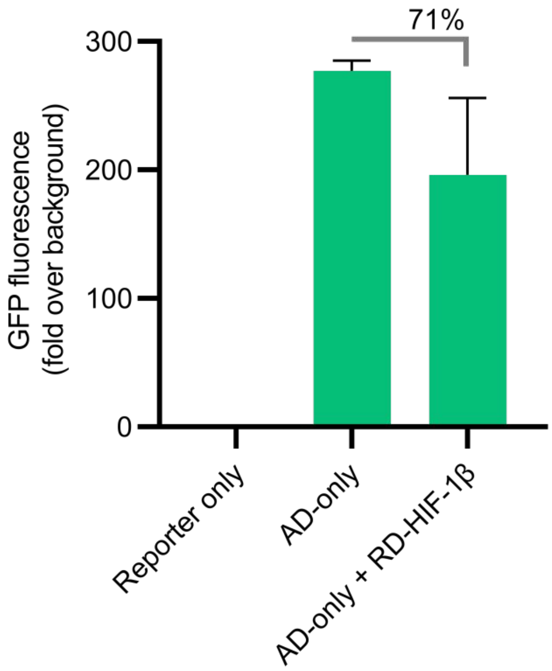

Figure S2. Biosensor reporter gene repression with HIF-1α removed as bait from the transactivator. Fluorescence (GFP) output is shown as maximal reporter gene (*lexAx4-pCYC1min-EGFP*) output (AD-only) compared to the repressed state (AD-only + RD-HIF-1β). Only 71% of the maximal fluorescence is achieved in the repressed state. Fluorescence was measured by flow cytometry after 16 h of culture, and bars represent mean values and error bars the range. AD = LexA-VPR and RD = Tup1.

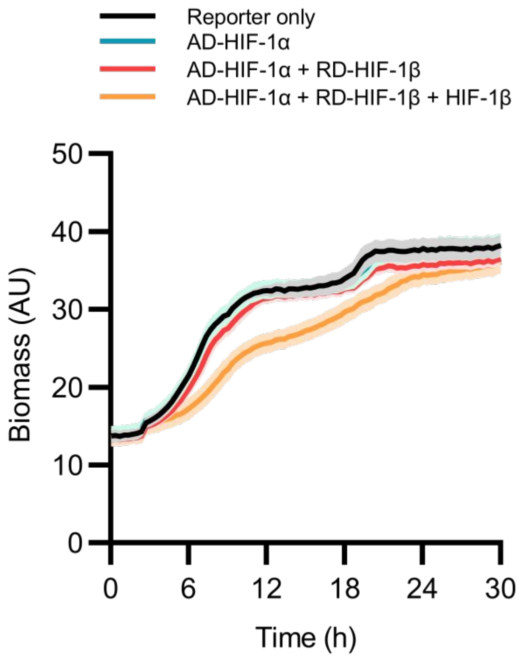

Figure S3. Growth over time for biosensor strains. Yeast strains were grown in a 48-well FlowerPlate in a BioLector (m2p-laboratories GmbH). Bars represent mean values and error bars the standard deviation. AD = LexA-VPR, RD = Tup1, AD-RD= LexA-VPR-Tup1.

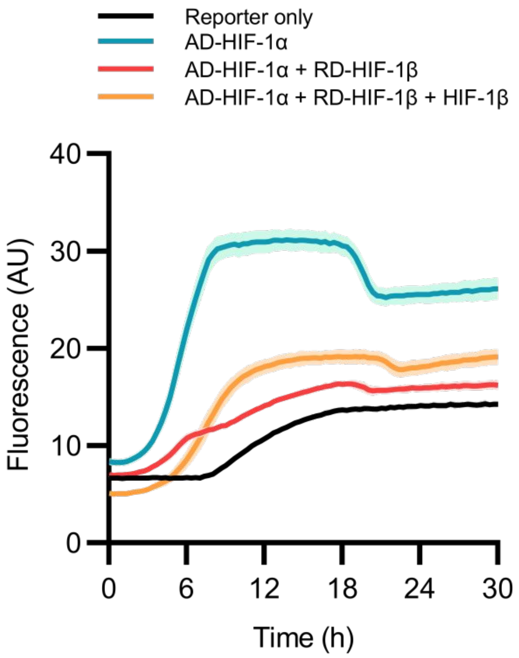

Figure S4. Fluorescence over time for biosensor strains. Yeast strains were grown in a 48-well FlowerPlate in a BioLector (m2p-laboratories GmbH). Bars represent mean values and error bars the standard deviation. AD = LexA-VPR, RD = Tup1, AD-RD= LexA-VPR-Tup1.

Table S1. Oligonucleotides used in this study to provide homology to genomic integration sites.

Site	Oligo ID	Sequence 5' - 3'
XII-1	XII-1_Hom_fwd<br>XII-1_Hom_rev	ccagatttggctttgattttggcatcggttcggttctttcattaagtcctcagcgagctc<br>gaactagttattaaggtatgtgcagttgattcacgggaagattaatgcctcagcactagt
X-2	X-2_ConLx_fwd<br>X-2_ConRx_rev	agagaaactcgcaggcaacttgctctcgaagtggtcacgtccctgaattcgcatctaga<br>atgggtaacgggatccctctgtgaggccgattatgcaggtgtactgcagtgcactagt
XI-5	XI-5_ConLx_fwd<br>XI-5_ConRx_rev	tgcaatgattacttaccaatgtgccataaactccgtgcaccaccctgaattcgcatctaga<br>ttgtgggcaattgggtgtactatgaagcagccaatagtatgtactgcagtgcactagt
XI-2	XI-2_ConLx_fwd<br>XI-2_ConRx_rev	taggcaaaagccaaggagcgtttgccatgaacttccacaaccctgaattcgcatctaga<br>acatctaaactttttaatatctgaaagcgctagtcgtgtgtgtactgcagtgcactagt
X-3	X-3_Hom_fwd<br>X-3_Hom_rev	acaataggcaagaagtaggcgagagccgacatacgagactattaagtcctcagcgagctc<br>cttttactagcatatcaatatccgtttcattgaaaagtggattaatgcctcagcactagt
